# Radio Resource Dimensioning for Low Delay Access in Licensed OFDMA IoT Networks †

**DOI:** 10.3390/s20247173

**Published:** 2020-12-15

**Authors:** Yi Yu, Lina Mroueh, Philippe Martins, Guillaume Vivier, Michel Terré

**Affiliations:** 1 Institut Supérieur d’Electronique de Paris, 92130 Issy Les Moulineaux, France; yi.yu@isep.fr; 2 Conservatoire National des Arts et des Métiers, 75003 Paris, France; michel.terre@cnam.fr; 3 Telecom Paris, 91120 Palaiseau, France; philippe.martins@telecom-paris.fr; 4 Sequans Communications, 92700 Colombes, France; gvivier@sequans.com

**Keywords:** LPWAN, licensed OFDMA-based IoT, resource planning, stochastic geometry

## Abstract

In this paper, we focus on the radio resource planning in the uplink of licensed Orthogonal Frequency Division Multiple Access (OFDMA) based Internet of Things (IoT) networks. The average behavior of the network is considered by assuming that active sensors and collectors are distributed according to independent random Poisson Point Process (PPP) marked by channel randomness. Our objective is to statistically determine the optimal total number of Radio Resources (RRs) required for a typical cell. On one hand, the allocated bandwidth should be sufficiently large to support the traffic of the devices and to guarantee a low access delay. On the other hand, the over-dimensioning is costly from an operator point of view and induces spectrum wastage. For this sake, we propose statistical tools derived from stochastic geometry to evaluate, adjust and adapt the allocated bandwidth according to the network parameters, namely the required Quality of Service (QoS) in terms of rate and access delay, the density of the active sensors, the collector intensities, the antenna configurations and the transmission modes. The optimal total number of RRs required for a typical cell is then calculated by jointly considering the constraints of low access delay, limited power per RR, target data rate and network outage probability. Different types of networks are considered including Single Input Single Output (SISO) systems, Single Input Multiple Output (SIMO) systems using antenna selection or Maximum Ratio Combiner (MRC), and Multiuser Multiple Input Multiple Output (MU-MIMO) systems using Zero-Forcing decoder.

## 1. Introduction

Cellular licensed IoT technology has been an emerging and evolving Low Power Wide Area (LPWA) technology which provides long range, low power and low cost connectivity for IoT devices [1]. It can be deployed in existing cellular networks from which it inherits many of the features that determine its behavior [2], such as Long Term Evolution (LTE) networks. With the booming development of Fifth Generation (5G) technology, evolutions of cellular IoT standards have been proposed and put into practice by 3rd Generation Partnership Project (3GPP) [3]. It will further promote the development of IoT technology. 5G technologies primarily include non-standalone and standalone technologies. The former is the early-drop technology that operators plan to connect new radio 5G base stations to the Fourth Generation (4G) core network until 5G connectivity migrates to the native 5G Core network mode which means shifts to standalone mode [4]. For the 4G and nonstandalone 5G connections, Orthogonal Frequency Division Multiple Access (OFDMA) technology can be employed to enable multi-carrier transmission and network access. OFDMA can take advantage of multiuser diversity and robustness to multipath fading for the uplink communications. Moreover, it provides more degrees of freedom for resource allocation and facilitates multiplexing and diversity gains [5,6]. Due to the limited spectrum of licensed IoT, as the network gradually scales up, one of the key issues we face is how to effectively use these resources to support large-scale IoT devices. In addition, the cost of IoT has been a major concern, and the improvement of spectrum efficiency can help reduce the cost of cellular IoT networks.

In this paper, we focus on the uplink of OFDMA-based cellular IoT networks with multiple antennas receiver. The average statistical behavior of the network is considered where the active sensors and collectors are randomly distributed in a given area according to a random Poisson Point Process (PPP). The randomness of the wireless channel is considered as a mark of the Poisson position and results from stochastic geometry using marked PPP as in [7,8,9,10,11,12,13,14,15,16,17,18,19] will be invoked. These tools were investigated in [20,21,22,23] to compute an upper-bound on the resource outage probability in a cellular network considering random PPP marked by the random fading. Unlike the previous contributions in [20,21,22,23] where the network is considered as noise limited, this study is more general as it takes into account the impact of interference on the statistical dimensioning. In [24], our dimensioning model is restricted to the single user case with multiple antennas receivers, in which only the receiver diversity, namely, the antenna selection technique, is performed without exploiting the spatial multiplexing gain. This paper generalizes our previous contribution in [24] by considering MU-MIMO schemes where multiple users can be scheduled over one RR. It introduces indeed a new criterion for performing dimensioning based upon low delay access. We also provide a comparison between the energy consumption of the different considered transmission modes.

We consider first single-user (SU) communication with receiver diversity transmission modes such as antenna selection considered in [24] and the Maximum Ratio Combining (MRC). Next, we consider multiuser (MU) transmission where a distance-based scheduling algorithm is proposed with neighboring sensors nodes that are scheduled on the same RR. The interuser interferences are canceled using a Zero-Forcing decoder. The remaining diversity when not all the degrees of freedom are consumed is extracted using a MRC. The number of RR required by a single node in the SU case of by a group of nodes in the MU case is limited by the lowest Modulation and Coding Scheme (MCS). We define the network outage as the event that occurs when the number of request RRs exceed the number of the available ones. Whenever this event occurs, the sensor node has to delay its transmission to the next time transmission interval (TTI). Our goal is to determine the number of required RR to be allocated at the network side depending on the network load and the collector density in order to guarantee that the average delay access does not exceed a preset threshold.

The rest of the paper is organized as follows. We introduce in Section 2 the network model and its properties. In Section 3, we review first the dimensioning concentration inequality used in [21,22,23,24] that provides an upper-bound on the network outage probability and hence on the number of required RR. This upper-bounds depends on the average total number of required RR that we computed for the single-user case as in [24] and for the multiuser case that we introduce in this paper. We also characterize in each case the power distribution. The results presented in this section are general and are not related to the transmission mode fading distribution observed by the collector or the statistical behavior of interference that are characterized in Section 4. Numerical results are provided in Section 5 to compare the transmission mode in terms of required RRs and energy consumption in function of the collectors density and the average required delay. Finally, Section 6 concludes the paper.

## 2. IoT Network Model

We consider a sensor network in which a random number of active nodes sensors and collectors are distributed in a given area A according to two independent homogeneous PPP with intensities $\lambda_{a}$ and $\lambda_{b}$. We assume that the sensors are equipped with a single antenna and that the collector is equipped with $n_{r}$ receiver antennas. We assume that a sensor is active $n_{a}$ times per day, the mean service time is $\nu^{-1}$ (s) and the interarrival rate is $\frac{n_{a}}{24\times 60\times 60}\;\rho$ per second and per km^2^. The active sensors nodes form then a spatial PPP $\Phi_{a}$ with intensity

(1) $$\lambda_{a}=\frac{n_{a}}{24\times 60\times 60}\rho \nu^{-1}.$$

The frequency reuse pattern in the network is equal to 1. At a given collector situated at $y_{0}\in \Phi_{b}$, the received power on a given RR from a sensor $x\in \Phi_{a}$ transmitting with power $P_{\mathrm{RR}}$ (mW per RR) is computed as, $P_{r}\left(y_{0},x\right)=P_{\mathrm{RR}}\;\alpha \;{\left|y_{0}-x\right|}^{-\beta }\;A_{f}$ where α and β are respectively the attenuation factor (that includes the average shadowing) and the path-loss exponent that are computed from the Okumura-Hata model, $A_{f}$ is the fading coefficient with distribution depending on the antenna configuration and the used transmission mode (single-user or multiuser). The sensor nodes are considered as static and the fading channel is considered as flat during the transmission. The Channel State Information (CSI) is only available at the receiver side and not at the transmitter side. Each sensor is connected to the collector on which the average received power (by averaging over the fading and the shadowing) is the highest. This is equivalent to connect the sensor to the nearest collector. Assuming a collector $y_{0}$, the set of the sensors connected to this latter is defined as $\Phi_{c}\left(y_{0}\right)=\{x\in \Phi :\forall y\in \Phi_{b}-\left\{y_{0}\right\}:|y_{0}-x|<|y-x|\}.$ Nodes transmitting in the same frequency band generate additive interference with power

(2) $$I\left(y_{0}\right)=\sum_{x_{i}\in \Phi_{I}\left(y_{0}\right)}P_{\mathrm{RR}}\alpha {\left|y_{0}-x_{i}\right|}^{-\beta }A_{f,i},$$

with

(3) $$\Phi_{I}\left(y_{0}\right)=\cup_{y\in \Phi_{b},y\neq y_{0}}\left\{x_{i}:x_{i}\;\mathrm{randomly}\;\mathrm{selected}\;\mathrm{in}\;\Phi_{c}\left(y\right)\right\}.$$

The received SINR at the given collector $y_{0}$ is,

(4) $$\mathrm{SIN}R\left(y_{0},x\right)=\frac{P_{\mathrm{RR}}\;\alpha \;{\left|y_{0}-x\right|}^{-\beta }\;A_{f}}{P_{n}+I\left(y_{0}\right)},$$

with $P_{n}$ being the random exponential noise power with mean power of ${\bar{P}}_{n}=KTB$ where *K* is the Boltzmann’s constant $K=1.379\times 10^{-23}$ W Hz^−1^ K^−1^, *T* the absolute temperature in kelvins T=290 K and *B* the bandwidth. The power of the random exponential noise power is characterized by its Laplace transform as,

(5) $$L_{P_{n}}\left(s\right)=E\left[e^{-sN}\right]=\frac{1}{s{\bar{P}}_{n}+1}.$$

**Figure 1:**
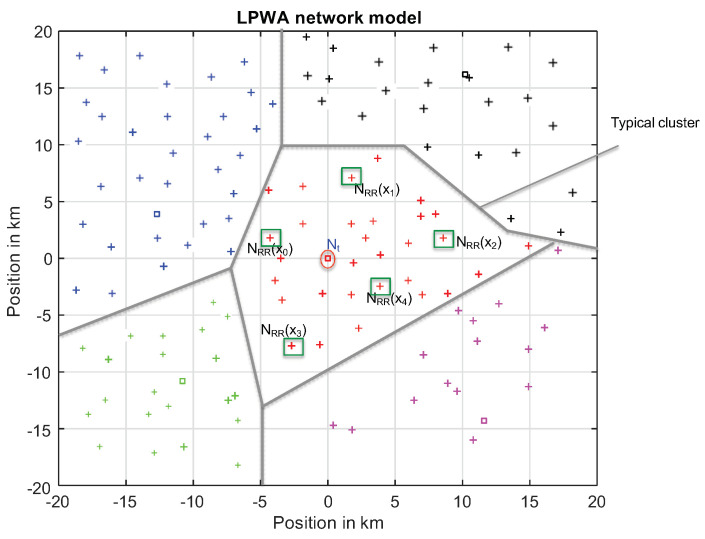
Network model: typical cell and active nodes.

Due to Slivnyak-Mecke Theorem in [8], the statistical behavior in the PPP remains unchanged when adding a collector at the center of this region. This defines the typical cell centered at the origin $\Phi_{c}\left(0\right)$ as illustrated in Figure 1. The typical cell properties are reviewed in Proposition 1.

**Proposition** **1** (Typical cell properties)**.** *The typical cell average number of sensors is,*

(6) $$N_{s}≜E\left[\sum_{x\in \Phi_{a}}1_{\left\{x\in \Phi_{c}\left(0\right)\right\}}\right]=\frac{\lambda_{a}}{\lambda_{b}}$$

*The probability distribution function of r=|x| the distances between $x\in \Phi_{c}\left(0\right)$ and the o collector is,*

(7) $$f\left(r\right)=2\pi \lambda_{b}\exp\left(-\lambda_{b}\pi r^{2}\right).$$

*The average radius of the typical cell is $1/(2\sqrt{\lambda_{b}})$.*

**Proof.**  We provide for completeness the proof in Appendix A. □

## 3. Proposed Statistical Dimensioning Model

Given the density of collectors in the network, we propose a statistical method for resource planning in the uplink of cellular IoT networks. We assume the network access is an OFDMA and consider only the Narrowband Physical Uplink Shared Channel (NPUSCH) which carries the uplink user data and uplink control information. In an NB-IoT network or LTE-M, identical to the LTE network, the smallest block of radio resource elements that can be invoked is called the Resource Block (RB) or Radio Resource Block (RRB). It contains several OFDM symbols. Each RR corresponds to 180 kHz in the frequency domain and 0.5 ms in the time domain. In the 5G, different numerologies were defined to reduce the RR duration and hence the latency by increasing the RR bandwidth. In the following, the RR corresponds to the LTE resource block and the RR with numerology 0 in the 5G terminology. For the single-user and multiuser cases, our main objective here is to statistically determine the total number of required RRs to minimize the occurrence of the network outage event. When the total number of RRs required is larger than the number of RRs available on the collector side, this is considered as an outage event.

### 3.1. Dimensioning Objectives in a Typical Cell

In the typical cell illustrated in Figure 1, the collector o allocates according to the level of the received SINR, 1 to $N_{\max}$ RRs to the sensor node in order to achieve its target data rate $C_{0}$. The required number of RRs is,

(8) $$N_{\mathrm{RR}}\left(x,A_{f},I,P_{n}\right)=\sum_{k=1}^{N_{\max}}k\times {𝟙}_{\left\{\mathrm{SINR}\left(x\right)\;\in \;[\gamma_{k}\;;\;\gamma_{k-1}]\right\}}.$$

with $\gamma_{k}$ being the threshold SINR required to achieve a target rate of $C_{0}/k$ within a single RR and $\gamma_{0}>\gamma_{1}>\cdots >\gamma_{N_{\max}}$ and $\gamma_{0}\to \infty$. Note that $\gamma_{0}$ (respectively $\gamma_{N_{\max}}$) is the threshold SINR to decode the highest (respectively lowest) Modulation and Coding (MCS) scheme. If $\mathrm{SINR}\left(0,x\right)<\gamma_{N_{\max}}$, the sensor will not be able to decode the lowest MCS and the collector does not attribute any RR to this user. This event of having insufficient SINR occurs with probability, we intentionally do not refer to this probability as outage to avoid confusion with the network outage probability.

(9) $$P_{\mathrm{off}}=\mathrm{Prob}\left\{\mathrm{SINR}\left(0,x\right)<\gamma_{N_{\max}}\right\}.$$

By setting

(10) $$A_{f,k}=\gamma_{k}\alpha^{-1}r^{\beta }\left(P_{n}+I\right),\;\forall 1\leq k\leq N_{\max},$$

with $A_{f,0}=\infty$ the threshold fading to achieve a rate $C_{k}\in \left[C_{0}/k\;;\;C_{0}/\left(k-1\right)\right]$ over one RR, the number of RR can be rewritten as,

(11) $$N_{\mathrm{RR}}\left(x,A_{f},I,P_{n}\right)=\sum_{k=1}^{N_{\max}}k\times {𝟙}_{\left\{A_{f,k}\leq A_{f}\leq A_{f,k-1}\right\}}.$$

The total of required RR in this typical cell depicted in Figure 1 is,

(12) $$N_{RR,t}\left(0\right)=\sum_{x\in \Phi_{c}\left(y_{0}\right)}N_{RR}\left(x,A_{f},I,P_{n}\right).$$

The network is in outage if,

(13) $$P_{\mathrm{out},c}\left(N_{t}\right)=\mathrm{Prob}\left\{N_{RR,t}\left(0\right)>N_{t}\right\}.$$

In order to ensure an optimized network dimensioning, the number of total radio resources $N_{t}$ that ensure a network outage probability of $p_{\mathrm{th},n}$, should be found. Using the concentration inequality, the typical cell outage probability is upper-bounded by,

(14) $$P_{\mathrm{out},c}\left(N_{t}\right)\leq P_{\sup}\left(N_{t}\right),$$

where

(15) $$P_{\sup}\left(N_{t}\right)=\exp\left(-\frac{v_{N}}{N_{\max}^{2}}g\left(\frac{N_{\max}\left(N_{t}-m_{N}\right)}{v_{N}}\right)\right),$$

with

(16) $$\begin{array}{ccc} m_{N} & = & E\left[\sum_{x\in \Phi_{c}\left(o\right)}N_{RR}\left(x\right)\right], \end{array}$$

(17) $$\begin{array}{ccc} v_{N} & = & E\left[\sum_{x\in \Phi_{c}\left(o\right)}N_{RR}^{2}\left(x\right)\right], \end{array}$$

the function g(t)=(1+t)log(1+t)−t and $N_{t}>m_{N}$. By setting a threshold $p_{th,n}$ on the network outage,

$$\begin{array}{ccc} N_{t} & = & m_{N}+\frac{v_{N}}{N_{\max}}g^{-1}\left(\frac{N_{\max}^{2}}{v_{n}}\log\left(\frac{1}{p_{\mathrm{th},n}}\right)\right). \end{array}$$

### 3.2. Average Delay and Choice of the Network Threshold

In this subsection, we compute the average delay to connect the sensor device to the network independently of its transmitting rate. This average delay should not exceed a maximal delay $\tau_{\max}$ that we assume proportional to the Time Transmission Interval (TTI). As long as the sensor is not accepted by the network due to the lack of resource, a new trial will be performed after TTI. We assume that the probabilities of being rejected after i×TTI are independent and are equal to ${\left(P_{\mathrm{out},c}\left(N_{t}\right)\right)}^{i}$. The average delay to access to the network is hence,

(18) $$\bar{\tau }=\left(\sum_{i=0}^{\infty }i\times \mathrm{TTI}\times {\left(P_{\mathrm{out},c}\left(N_{t}\right)\right)}^{i}\right)\leq \left(\sum_{i=0}^{\infty }i\times \mathrm{TTI}\times p_{th,n}^{i}\right).$$

This expression can be simplified to,

(19) $$\bar{\tau }\leq \mathrm{TTI}\frac{p_{th,n}}{{\left(1-p_{th,n}\right)}^{2}}.$$

By choosing $p_{th,n}$ such that

(20) $$\mathrm{TTI}\frac{p_{th,n}}{{\left(1-p_{th,n}\right)}^{2}}≜\tau_{\max},$$

we make sure that the average delay in the network does not exceed $\tau_{\max}$. By letting $\kappa =\tau_{\max}/\mathrm{TTI}$, the network threshold is,

(21) $$p_{th,n}=\frac{\left(2\kappa +1\right)-\sqrt{1+4\kappa }}{2\kappa }<1.$$

### 3.3. Expressions of $m_{N}$ and $v_{N}$

In this subsection, we derive the expressions of $m_{N}$ and $v_{N}$ in (16) and (17) considering the single-user and multiuser cases. We recall that all sensor devices are equipped with a single antenna and the number of antennas at the collector is $n_{r}$. The distribution of the fading coefficient in (4) will be specified in the next section depending on the antenna configuration and transmission mode.

#### 3.3.1. Single-User Case

**Proposition** **2** (Single-user case)**.** *The expressions of $m_{N}$ and $v_{N}$ are,*

(22) $$\begin{array}{ccc} m_{N} & = & N_{s}\;\times \sum_{k=1}^{N_{\max}}k\times E_{r}\;E_{I,P_{n}}\;E_{A_{f}}\left[{𝟙}_{\left\{A_{f,k}\leq A_{f}<A_{f,k-1}\right\}}\right] \end{array}$$

(23) $$\begin{array}{ccc} v_{N} & = & N_{s}\;\times \sum_{k=1}^{N_{\max}}k^{2}\times E_{r}\;E_{I,P_{n}}\;E_{A_{f}}\left[{𝟙}_{\left\{A_{f,k}\leq A_{f}<A_{f,k-1}\right\}}\right] \end{array}$$

*with $N_{s}=\lambda_{a}/\lambda_{b}$ the average number of sensors as shown in (6), and $E_{r}\left[\left(.\right)\right]=\int_{0}^{\infty }\left(.\right)f\left(r\right)dr$ with f(r) defined in (7).*

**Proof.**  Please refer to Appendix B. □

Note that (22) can be interpreted as following: $N_{s}$ is the average number of sensor in the typical cell; each sensor requires each 1 to $N_{\max}$ RRs depending on their distance to the collector, the channel conditions and the additive interference and noise.

#### 3.3.2. Multiuser Case

**Figure 2:**
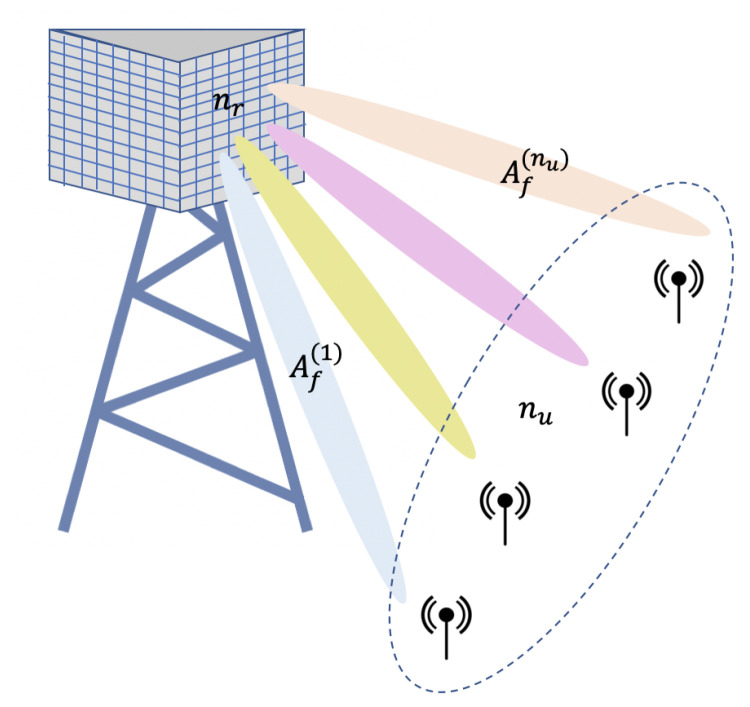
MU-MIMO: $n_{u}$ users are simultaneously scheduled on the same radio-resource.

In the multiuser case, we assume that each RR is shared by $n_{u}$ users simultaneously as depicted in Figure 2. To cancel the interuser interference, the collectors uses a Zero-Forcing (ZF) decoder. The equivalent fading observed by each sensor *j* device is denoted by $A_{f}^{\left(j\right)}$ having a distribution that will be specified in the next section.

**Figure 3:**
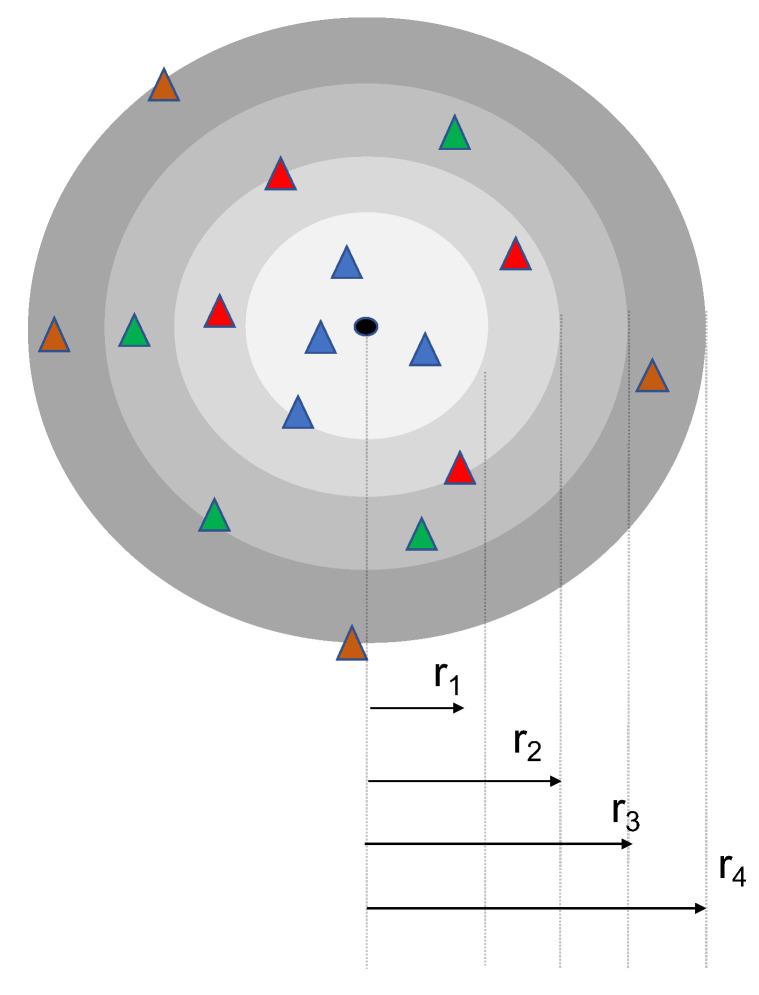
Illustration of the scheduling with $n_{u}=4$. The number of RR is adjusted with respect to the furthest node in each group, to say *i*, situated at distance $r_{i}$ corresponding to the radius of the ball containing $in_{u}-1$ nodes.Distance based multiuser scheduling scheme

Distance based multiuser scheduling scheme: In this scheme, the active users are sorted according to their proximity to the collector into different groups of $n_{u}$ users, each independently of their fading. As shown in Figure 3, the first group contains the $n_{u}$ nearest neighbor to the typical collector, the second group the ${\left(n_{u}+1\right)}^{\mathrm{th}}$ to $2n_{u}^{\mathrm{th}}$ collector neighbor and so on. We adjust the number of allocated RRs to the furthest user from the collector in each group independently of the fading coefficient experienced by other users. The furthest node *x* in each group *i* is the $i\times n_{u}^{\mathrm{th}}$ neighbor of the collector and it corresponds to the radius of the ball B(|x|) with radius |x| containing $i\times n_{u}$ nodes.

**Proposition** **3** (Multiuser case)**.** *The expressions of $m_{N}$ and $v_{N}$ are*

(24) $$\begin{array}{ccc} m_{N} & = & N_{s}\times \sum_{k=1}^{N_{\max}}k\times E_{r}\;E_{I,P_{n}}\;E_{A_{f}}\left[{𝟙}_{\left\{A_{f,k}\leq A_{f}<A_{f,k-1}\right\}}\sum_{i\in N^{*}}\mathrm{Prob}\left\{\left|B\left(r\right)\right|=in_{u}-1\right\}\right] \end{array}$$

(25) $$\begin{array}{ccc} v_{N} & = & N_{s}\times \sum_{k=1}^{N_{\max}}k^{2}\times E_{r}\;E_{I,P_{n}}\;E_{A_{f}}\left[{𝟙}_{\left\{A_{f,k}\leq A_{f}<A_{f,k-1}\right\}}\sum_{i\in N^{*}}\mathrm{Prob}\left\{\left|B\left(r\right)\right|=in_{u}-1\right\}\right] \end{array}$$

*where B(r) is the ball with radius r, and*

(26) $$\sum_{i\in N^{*}}\mathrm{Prob}\left\{\left|B\left(r\right)\right|=in_{u}-1\right\}=\exp\left(-\lambda_{a}\pi r^{2}\right)\sum_{i\in N^{*}}\frac{{\left(\pi \lambda_{a}r^{2}\right)}^{\left(in_{u}-1\right)}}{(in_{u}-1)!}.$$

with $N^{*}$ being the set of nonzero positive integers. The distribution of r in the typical cell is given by f(r) defined in (7).

**Proof.**  Please refer to Appendix C. □

Note that Proposition 3 is a generalization of Proposition 2 with $n_{u}=1$. For $n_{u}=1$,

(27) $$\sum_{i\in N^{*}}\frac{{\left(\pi \lambda_{a}r^{2}\right)}^{\left(i-1\right)}}{(i-1)!}=\exp\left(-\lambda_{a}\pi r^{2}\right)$$

and the expressions of $m_{N}$ in (24) becomes equal to (22).

### 3.4. Distribution of the Sensor Power Consumption

We assume that the transmitted power is fixed to $P_{\mathrm{RR}}$ per RR and the total power over $N_{\max}$ RRs does not exceed $P_{\max}=N_{\max}P_{\mathrm{RR}}$. The power is then a discrete random variable and its Probability Mass Function (PMF) is given in Proposition 4.

**Proposition** **4** (Total sensor power PMF)**.** *For the general case, the sensor power PMF is,*

(28) $$\mathrm{Prob}\left\{P_{t}=kP_{\mathrm{RR}}\right\}=\frac{N_{s}}{N_{g}}\;E_{r}\;E_{I,P_{n}}\;E_{A_{f}}\left[{𝟙}_{\left\{A_{f,k}\leq A_{f}<A_{f,k-1}\right\}}\sum_{i\in N^{*}}\mathrm{Prob}\left\{\left|B\left(r\right)\right|=in_{u}-1\right\}\right],$$

*with $1\leq k\leq N_{\max}$, $N_{g}$ the number of the groups of $n_{u}$ sensors in the typical cell such that,*

(29) $$N_{g}=\int_{0}^{\infty }2\pi \lambda_{a}r\exp\left(-\lambda_{b}\pi r^{2}\right)\exp\left(-\lambda_{a}\pi r^{2}\right)\sum_{i\in N^{*}}\frac{{\left(\pi \lambda_{a}r^{2}\right)}^{\left(in_{u}-1\right)}}{(in_{u}-1)!}dr$$

*and $N_{s}=\lambda_{a}/\lambda_{b}$.*

**Proof.**  The proof is similar to Proposition 3 and the PMF can be obtained by averaging over the whole power in the typical cell normalized by the size of this latter. In each ring delimited by the $\left(i-1\right)\times n_{u}^{\mathrm{th}}$ and $i\times n_{u}^{\mathrm{th}}$ neighbor, the $n_{u}$ sensors adjust their power to the the $i\times n_{u}^{\mathrm{th}}$ furthest neighbor. □

## 4. Dimensioning Tools: Interference and Fading Characterization

In this section, we characterize the fading and interference distribution required to compute the average number of RR considering different antenna configuration using Single-User and Multiuser (MU-MIMO) communications.

### 4.1. Single-User: Case of Single Antenna Receiver

In this subsection, we review first from [9] the characterization of the Laplace transform of interference and the distribution of the fading in the SISO case averaged on the random noise plus interference.

#### 4.1.1. Interference Laplace Transform

Considering a SISO antenna configuration, the Laplace transform of the interference is,

(30) $$L_{I}\left(s\right)=E\left[e^{-sI}\right]=E\left[\prod_{x_{i}\in \Phi_{I}}e^{-sP_{\mathrm{RR}}\;\alpha {\left|x_{i}\right|}^{-\beta }A_{f,i}}\right],$$

in which $\Phi_{I}$ is the set of the interfering nodes that transmit on the same RR as shown in (3). The set of interfering nodes $\Phi_{I}$ cannot be considered as homogeneous PPP and its exact statistical distribution is generally a difficult problem to model. For this, we use the same approximation proposed in [7] that is shown to almost capture the statistical behavior of the interference field. Let $d_{i}$ be the distance between the interferer and the intended collector. The probability of finding at least one collector in the ball with radius $d_{i}$ centered at the interferer is $\left(1-e^{-\lambda_{b}\pi d_{i}^{2}}\right)$. In this case, a node at distance $d_{i}$ from the intended collector is considered as interfering with probability of $\left(1-e^{-\lambda_{b}\pi d_{i}^{2}}\right)$. It was shown in [7] that the effective interference observed at the tagged collector can be approximately modeled as a nonhomogeneous PPP with intensity $\lambda_{b}\left(1-e^{-\lambda_{b}\pi d_{i}^{2}}\right)$. Considering exponential power fading attenuation coefficients, the interference Laplace transform from (30) for the SISO case is,

(31) $$L_{I}\left(s\right)=E\left[\prod_{x_{i}\in \Phi_{I}}\frac{1}{1+sP_{\mathrm{RR}}\;\alpha \;{\left|x_{i}\right|}^{-\beta }}\right].$$

Using the Probability Generating Functional (PGFL) property, the Laplace transform is then,

(32) $$L_{I}^{\mathrm{SISO}}\left(s\right)\approx L_{I}\left(s\right)≜\exp\left(-2\pi \lambda_{b}\int_{0}^{\infty }\left[\frac{sP_{\mathrm{RR}}\;\alpha \;u^{-\beta }}{1+sP_{\mathrm{RR}}\;\alpha \;u^{-\beta }}\right]\left(1-e^{-\lambda_{b}\pi u^{2}}\right)udu\right).$$

#### 4.1.2. Average Fading Distribution

**Proposition** **5** (SISO case)**.** *Given a node position r=|x|, the SISO fading distribution averaged on the random noise and interference,*

(33) $$\begin{array}{ccc} E_{I,P_{n}}\;E_{A_{f}}\left[{𝟙}_{\left\{A_{f,k}\leq A_{f}<A_{f,k-1}\right\}}\right] & = & L_{P_{n}}\left(s_{k}\right)L_{I}\left(s_{k}\right)-L_{P_{n}}\left(s_{k-1}\right)L_{I}\left(s_{k-1}\right), \end{array}$$

*with $s_{k}=\gamma_{k}P_{\mathrm{RR}}^{-1}\alpha^{-1}r^{\beta }$.*

**Proof.**  Please refer to Appendix D. □

### 4.2. Single-User Case with Multiantenna Receiver

**Figure 4:**
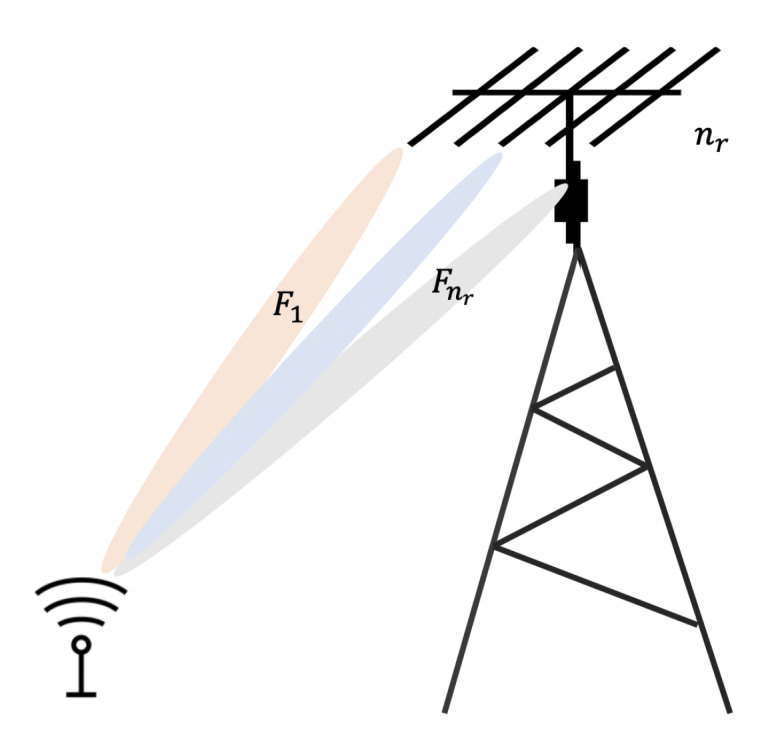
Receiver diversity with SIMO configuration.

We assume a SIMO configuration depicted in Figure 4 which $n_{r}$ antennas are used at the receiver and only a single antenna at each sensor node. Let $F_{1},\ldots ,F_{n_{r}}$ denote the fading coefficients between the sensor device and the $n_{r}$ receive antennas. Assume that the receiver antennas are sufficiently separated to assume that the random exponential fading coefficients are independent.

Two transmission modes are considered: the first transmission is the antenna selection in which the transmission is performed on the path with highest fading coefficient. The second transmission is the Maximum Ratio Combiner (MRC), in which the received signals at the different antenna are combined.

#### 4.2.1. Antenna Selection

Given a node position r=|x|, the SIMO fading distribution with antenna selection averaged on the random noise and interference, $A_{f}=\max\left(F_{1},\ldots ,F_{n_{r}}\right)$ is distributed as the maximum between $n_{r}$ i.i.d exponential variables. The CDF function is then,

(34) Prob{Af≤u}=(1−e−u)nr=∑p=0nr(−1)ppnre−pu.

**Proposition** **6** (Antenna selection)**.** *The interference Laplace transform with antenna selection is identical to the SISO case in (32),*

(35) $$L_{I}^{\mathrm{sel}}\left(s\right)=L_{I}\left(s\right).$$

*Given a node position r=|x|, the SIMO fading distribution with MRC averaged on the random noise and interference,*

(36) EI,PnEAf[𝟙{Af,k≤Af<Af,k−1}]=∑p=0nr(−1)ppnrLPn(psk−1)LI(psk−1)−LPn(psk)LI(psk)

**Proof.**  The interfering signal is received on a random fading coefficient that is exponentially distributed as in the SISO case. The identify in (36) can be deduced from the distribution of $A_{f}$ given in (34). □

#### 4.2.2. Maximum Ratio Combiner (MRC)

Using a MRC, the equivalent fading coefficient $A_{f}=F_{1}+\ldots +F_{n_{r}}$ is the sum of $n_{r}$ random exponential i.i.d. random variables and it is distributed as chi-squared random variable with $2n_{r}$ degrees of freedom. The CDF of $A_{f}$ is,

(37) $$\mathrm{Prob}\left\{A_{f}\leq u\right\}=1-\sum_{p=0}^{n_{r}-1}\frac{1}{p!}u^{p}e^{-u}.$$

**Proposition** **7** (MRC decoder)**.** *The interference Laplace transform with MRC is identical to the SISO case in (32)*

(38) $$L_{I}^{\mathrm{MRC}}\left(s\right)=L_{I}\left(s\right).$$

*Given a node position r=|x|, the SIMO fading distribution with MRC averaged on the random noise and interference,*

(39) $$E_{I,P_{n}}\;E_{A_{f}}\left[{𝟙}_{\left\{A_{f,k}\leq A_{f}<A_{f,k-1}\right\}}\right]=\sum_{p=0}^{n_{r}-1}\frac{1}{p!}\left(\Xi_{p}\left(s_{k}\right)-\Xi_{p}\left(s_{k-1}\right)\right)$$

*with*

(40) Ξp(s)=(−1)p∑j=0pspjpdjLPn(s)dsjdp−jLI(s)dsp−j,

where $\frac{d^{j}f\left(s\right)}{ds^{j}}$ is the derivative of order j of the function f(s) for $j\in N^{*}$ and $\frac{d^{0}f\left(s\right)}{ds^{0}}=f\left(s\right)$.

**Proof.**  The proof of this proposition is detailed in Appendix E. □

### 4.3. Multiuser with Multiantenna Receiver

In order to cancel the multiuser interference, a Zero-Forcing decoder is used by projecting the $n_{r}\times 1$ received signal on the kernel of the space formed by the $n_{r}\times 1$ fading vectors of the $\left(n_{u}-1\right)$ other scheduled users. As stated by [25], the orthogonality constraints consume $\left(n_{r}-n_{u}+1\right)$ degrees of freedom. The receiver diversity for each sensor device is limited then to $\left(n_{r}-n_{u}+1\right)$.

To extract this diversity, we assume that the ZF decoder is followed by a MRC that combines the $\left(n_{r}-n_{u}+1\right)$ received observation. The fading coefficient $A_{f}^{\left(j\right)}$ observed by each sensor in Figure 2 is then chi-squared distributed with $2(n_{r}-n_{u}+1)$ degrees of freedom.

**Proposition** **8** (MU case with ZF-MRC decoder)**.** *For the MU-MIMO case with ZF decoder followed by a MRC, the equivalent fading distribution averaged on the random noise and interference,*

(41) $$E_{I,P_{n}}\;E_{A_{f}}\left[{𝟙}_{\left\{A_{f,k}\leq A_{f}<A_{f,k-1}\right\}}\right]=\sum_{p=0}^{n_{r}-n_{u}}\frac{1}{p!}\left(\Xi_{p}\left(s_{k}\right)-\Xi_{p}\left(s_{k-1}\right)\right).$$

*The interference Laplace transform is approximated by,*

(42) $$L_{I}^{\mathrm{MU}}\left(s\right)\approx L_{I}\left(n_{u}s\right).$$

**Proof.**  For the multiuser case, $n_{u}$ users in each cell transmit in the same RR. The interference becomes higher than the SISO case. As all neighboring users are multiplexed on the same RR, we approximate the sum of interference coming from the group of $n_{u}$ users by an interfering signal generated by a random interferer transmitting with $n_{u}P_{\mathrm{RR}}$. We have validated this approximation using numerical results as it will be shown in the next section.

The rest of the proof is similar to Proposition 7 considering $2(n_{r}-n_{u}+1)$ degrees of freedom. □

## 5. Numerical Results

In this section, we consider the uplink of a sensor network corresponding to the licensed IoT with parameters summarized in Table 1.

We assume that the density of nodes ρ=500 sensors per km^2^ transmitting on average once each half-an-hour, $n_{a}=48$ during 20 seconds. The active node density is then $\lambda_{a}=5.5$ sensors per km^2^. We consider a cellular network with ranges between 500 m to 1.5 km corresponding to a collector density of $\lambda_{b}=0.9$ nodes per km^2^ down to 0.1 nodes per km^2^ (the ranges are obtained with a confidence margin of 95%). The maximal power is limited to 14 dBm and is uniformly distributed among the allocated RRs. Table 2 gives the matching between the SINR range with the required number of RRs to achieve a target rate of $C_{0}=500$ bps. This data are derived from the Link Layer Simulation (LLS) provided in [26] on the Physical Uplink Shared Channel (PUSCH) of LTE-Cat M. To evaluate network performances, we consider a path-loss in an urban/suburban environment with $\alpha =10^{-14.1}$ and β=3.5. We consider the following antenna configurations: SISO, single SIMO with $n_{r}=8$, multiuser SIMO with $n_{r}=8$ and $n_{u}=2$ or $n_{u}=4$.

### 5.1. Accuracy of the Theoretical Model

Table 3 indicates the percentage difference between the mean number of RRs required in the typical cell derived by using our statistical dimensioning tools and the empirical network simulation results. The mean number of required RRs using former method is denoted as $m_{N}$, the simulation one is denoted as $m_{N,s}$. Consequently, the percentage difference is $\Delta m_{N}/m_{N,s}=\left|m_{N}-m_{N,s}\right|/m_{N,s}$. We consider different collector intensities (from 0.1 to 0.9 nodes/km^2^) and different antenna configurations as well as transmission modes. As presented in the Table 3, the comparison shows that the difference between the results obtained by our statistical tools and the simulated values is very small, which verifies the accuracy of our model and approach.

### 5.2. Average and Total Number of RR

**Figure 5:**
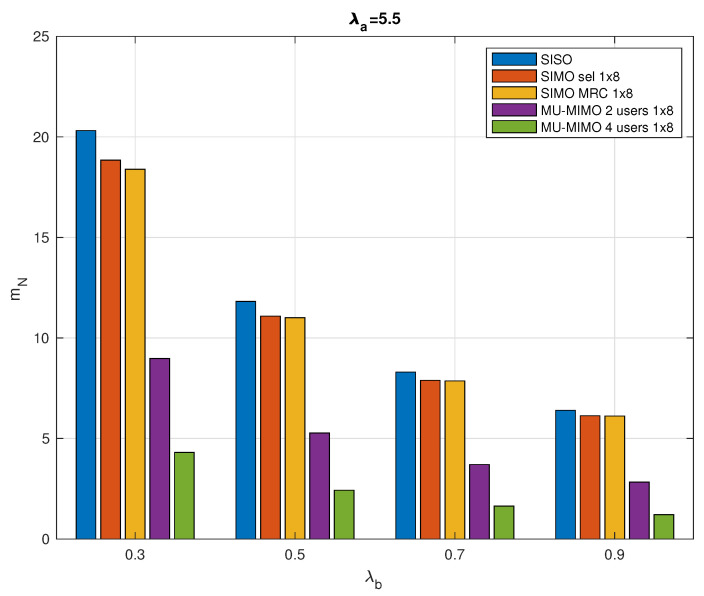
Mean number of required Radio Resources (RRs) in a typical cell considering $\lambda_{b}$ ranges from 0.3 to 0.9 nodes/${\mathrm{km}}^{2}$ with $\lambda_{a}=5.5$ nodes/${\mathrm{km}}^{2}$, $\tau_{max}=1$ ms.

Figure 5 further illustrates the specific value of $m_{N}$ considering different network configurations. As shown in the figure, for the given active sensor and collector intensities $\lambda_{a}$ and $\lambda_{b}$, respectively, assuming a maximum delay of 1 ms, the SISO system always requires the highest mean number of RRs.

With the use of SIMO and MU-MIMO, multiantenna techniques achieve higher data rates through increased spectral efficiency, with a corresponding reduction in the required RRs. The multiple receive antennas system has the potential to enhance signal robustness and increase system capacity. In particular, the MU-MIMO system significantly reduces the mean number of RRs required in a typical cluster. Meanwhile, as collector intensity $\lambda_{b}$ increases, the mean number of RRs required for various configurations decreases. When $\lambda_{b}$ increases, the radius of the typical cell will decrease, while the number of active sensor nodes per unit area remains the same, which means that each collector needs to serve fewer nodes in the cell and thus the number of RRs required decreases.

**Figure 6:**
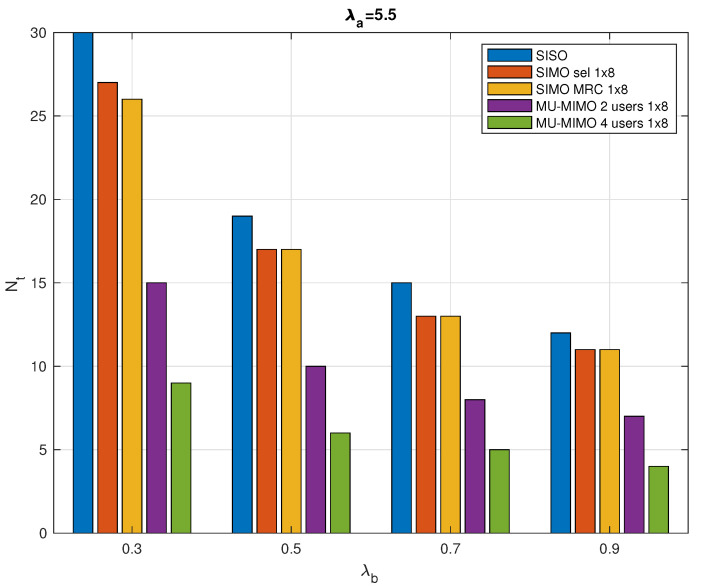
Total number of required RRs in a typical cell versus the collector intensity $\lambda_{b}$ with $\lambda_{a}=5.5$ nodes/${\mathrm{km}}^{2}$, $\tau_{max}=1$ ms.

Figure 6 presents the total number of required RRs in a typical cell versus the collector intensity. It maintains the same trend as in Figure 5, with MU-MIMO being the best performing case, followed by SIMO and then SISO.

### 5.3. Empirical Distribution and Actual Average Delay

**Figure 7:**
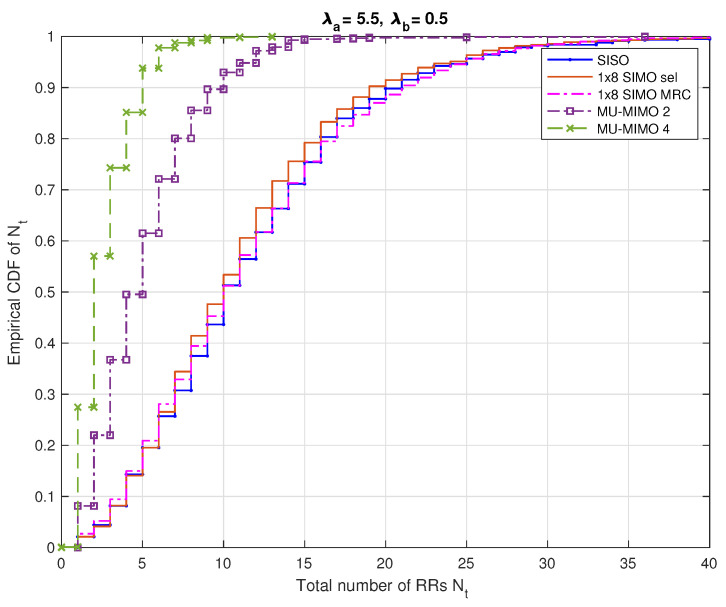
The empirical Cumulative Distribution Function (CDF) of $N_{t}$ total number of RRs required for the typical cell in which the maximal delay $\tau_{max}=1$ ms, $\lambda_{a}=5.5$ nodes/${\mathrm{km}}^{2}$ and $\lambda_{b}=0.5$ nodes/${\mathrm{km}}^{2}$.

Figure 7 illustrates the empirical Cumulative Distribution Function (CDF) of the total number $N_{t}$ of RRs required for the typical cell with $\lambda_{a}=5.5$ nodes/${\mathrm{km}}^{2}$ and $\lambda_{b}=0.5$ nodes/${\mathrm{km}}^{2}$.

For example, according to Figure 6, for the same value of $\lambda_{a}$, $\lambda_{b}$, we find that the $N_{t}$ value required for the SISO system is 19. In Figure 7, we can see that $N_{t}=19$ corresponds to a CDF value of 0.88. This means that there is a network outage probability of 1−0.88=0.12 meaning that the network cannot attributed RR to 12% of nodes in the typical cell. The corresponding average delay can be then computed using (19).

**Figure 8:**
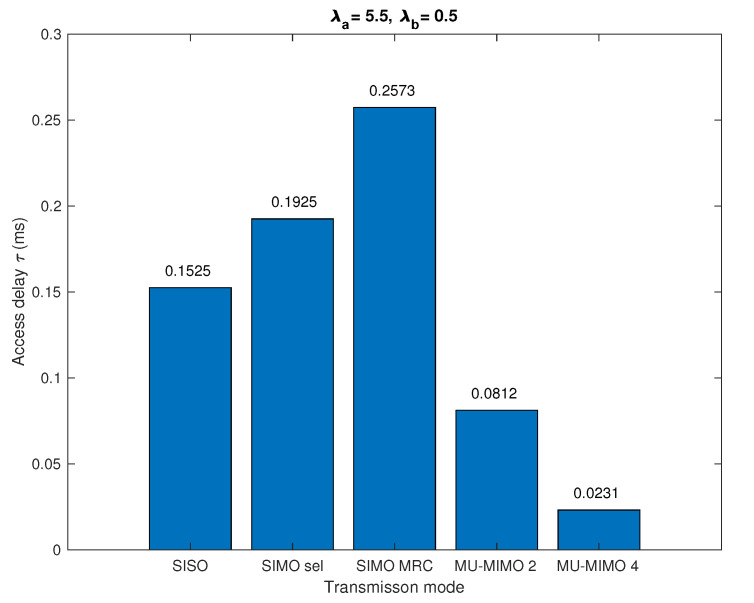
Low access delay τ with statistical dimensioning in which the maximal delay $\tau_{max}=1$ ms, $\lambda_{a}=5.5$ nodes/${\mathrm{km}}^{2}$ and $\lambda_{b}=0.5$ nodes/${\mathrm{km}}^{2}$.

Figure 8 show the actual average access delay corresponding to different antenna configurations and transmission modes for given intensities of active sensors and collectors $\lambda_{a}$, $\lambda_{b}$ and the maximum access delay $\tau_{max}=1$ ms. It can be seen from the figure that the average access delay τ is less than the maximum access delay $\tau_{max}$. SIMO with MRC transmission mode requires the longest access time because it has the lowest individual off probability, i.e., it serves the largest percentage of active nodes. The second highest access delay is the SIMO with antenna selection scheme. For the SISO configuration, the average access delay is relatively lower because in this mode, many nodes are in off state and not served. For the MU-MIMO system, each time there are $n_{u}$ nodes transmitting information simultaneously and being processed by the collector. The larger the $n_{u}$ is, the shorter the average access delay required.

### 5.4. Tolerated Delay and Overdimensioning

**Figure 9:**
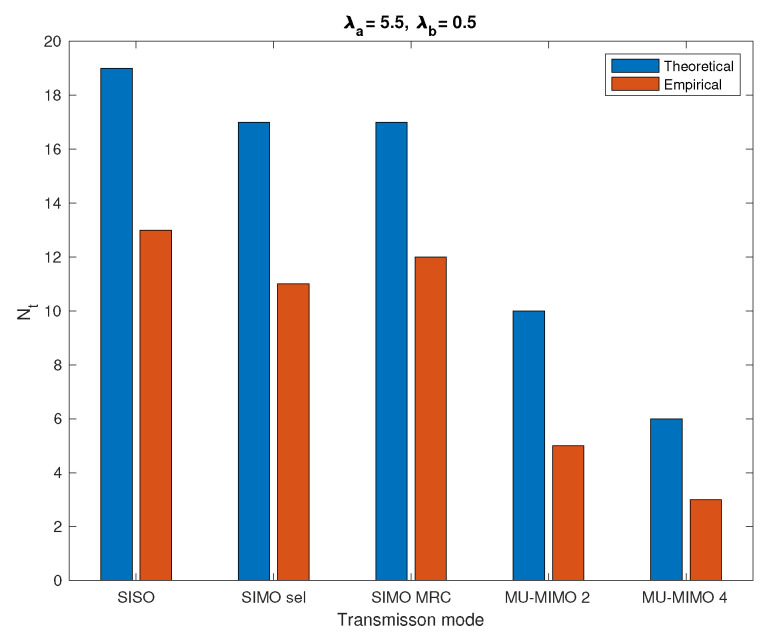
Comparison between theoretical and empirical $N_{t}$ values in which $\lambda_{a}=5.5$ nodes/${\mathrm{km}}^{2}$ and $\lambda_{b}=0.5$ nodes/${\mathrm{km}}^{2}$.

Figure 9 gives a comparison of the theoretical and empirical values of $N_{t}$ for a typical cell that guarantee an average delay of 1 ms.

From the figure, it can be seen that the $N_{t}$ derived by our statistical tool is always larger than the empirical $N_{t}$, i.e., it is overdimensioning. The excess in the radio-resource provided by the theoretical model ensures that the expected data rate is achieved and the access to the cellular IoT network does not exceed the predefined average access delay.

**Figure 10:**
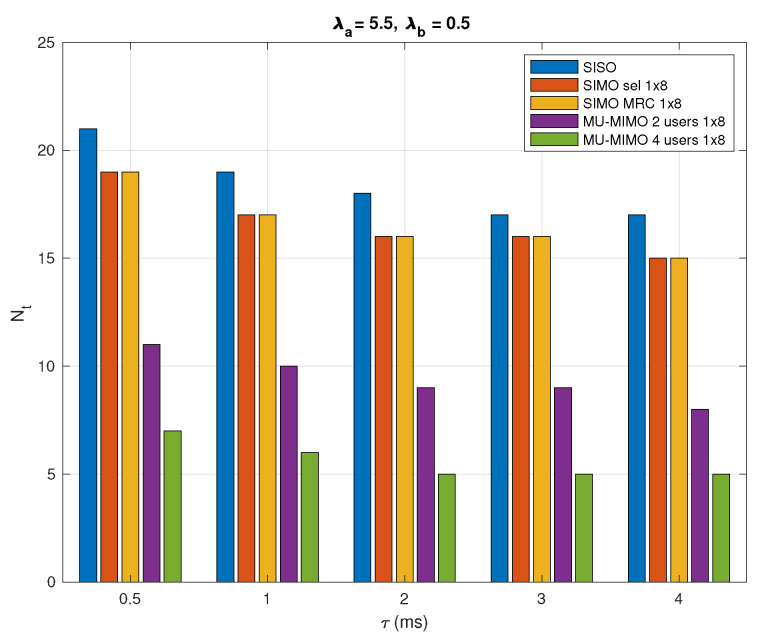
Total number of required RRs in a typical cell with respect to maximal access delay $\tau_{max}$ with $\lambda_{a}=5.5$ nodes/${\mathrm{km}}^{2}$ and $\lambda_{b}=0.5$ nodes/${\mathrm{km}}^{2}$.

In Figure 10, it shows the relationship between the total number of required RRs and the maximal access delay $\tau_{max}$ with $\lambda_{a}=5.5$ nodes/${\mathrm{km}}^{2}$ and $\lambda_{b}=0.5$ nodes/${\mathrm{km}}^{2}$. Assuming that $\tau_{max}$ ranges from 0.5 to 4 ms, a larger $\tau_{max}$ means that the node can wait longer for access to the network. The average access delay τ of the active nodes must not exceed the maximal delay $\tau \leq \tau_{max}$. In general, a lower total number of RRs is required to response to the demands of active sensor nodes with a larger $\tau_{max}$.

### 5.5. Individual Sensor off Probability

As mentioned in Section 3.1, when the SINR received on the collector side is below the SINR threshold $\gamma_{N_{max}}$, no RR will be assigned to this sensor. In other words, this active sensor is forced to be off in this trial. Table 4 illustrates the individual off probability in the typical cell according to the different antenna configurations, including SISO, 1×8 SIMO with antenna selection and MRC, 2 users and 4 users 1×8 MU-MIMO. From the table, we can see the 1×8 SIMO with MRC outperforms other modes in the individual off probability. Although it does not reduce the number of RRs required in the typical cell significantly compared to the SIMO with antenna selection or the MU-MIMO, it does reduce the sensor individual off probability, ensuring more sensors to be served. This result gives us a new way of thinking about resource planning, i.e., we need to take into account both the total number of RRs assigned to the network and the individual off probability to improve network service.

### 5.6. Power Distribution

In Table 5, for a given pair of $\lambda_{a}$ and $\lambda_{b}$, we elaborate the power distribution with the different transmission modes in the typical cell. The transmission power of each RR is fixed to $P_{\mathrm{RR}}=25/6$ mW (6.3 dBm), so the maximal transmission power is $N_{\max}P_{\mathrm{RR}}=25$ mW (14 dBm) with $N_{\max}=6$. For each transmission mode, Table 5 shows the specific percentage share of each transmit power. Where off represents a number of nodes to which no RR is assigned and therefore have a transmission power of 0 mW. From $P_{\mathrm{RR}}$ to $6P_{\mathrm{RR}}$ corresponds to the transmission power of the nodes that are assigned the corresponding number of 1 to 6 RRs, respectively. As marked in the light grey part of the table, it can be noticed that SIMO with MRC mode has the least number of off nodes, meanwhile with 99.92% of the nodes being transmitting information at $P_{\mathrm{RR}}$ power. Therefore, it has the best power distribution. MU-MIMO with $n_{u}=2$ also has a good power distribution, with only 0.05% of nodes off and 99.80% of nodes transmitting at $P_{\mathrm{RR}}$. SISO has the worst performance, with up to 3.97% of nodes unable to transmit information and only 90.76% of nodes transmitting at low power.

## 6. Conclusions

In this paper, we focus on licensed OFDMA-based IoT networks which corresponds to NB-IoT, LTE-M and to some extent 5G network using the numerology 0. We assume a wireless sensor network in which sensor nodes and collectors are distributed according a spatial PPP. A statistical method based on PPP is proposed to model and analyze the average behavior of the cellular IoT networks. This method is used for statistical resource planning to support the dominant uplink communications in the IoT networks and fulfill the network requirements in terms of limited transmission power per RR, low access delay, preset network outage probability and target data rate. Different antenna configurations and transmission modes are taken into account, e.g., SISO, SIMO, and MU-MIMO. Based on the preset tolerated average access delay and target data rate, the specific total number of RRs required for the typical cell is further calculated. Numerical results are finally given to assess our statistical analysis. We compare the theoretical results with the empirical simulation results in detail. The comparison shows that the results obtained from our statistical model are very close to the empirical results. Moreover, the results highlight and quantify the radio resources gain obtained by the receiver diversity techniques and the multiuser gains.

## Figures and Tables

**Table 1:** Network Parameters.

Parameters	Value
Intensity of active nodes	$\lambda_{a}=5.5$ nodes per km^2^
Intensity of collectors	$\lambda_{b}=0.1$ to 0.9 nodes per km^2^
Transmission power	$P_{\mathrm{RR}}=6.3$ dBm
RR per node	1 to 6 RRs
Configuration	SISO, single-user 1×8 SIMO,
	1×8 MU-MIMO with $n_{u}=2$ or 4
Okumura-Hata model	$\alpha =10^{-14.1}$, β=3.5
Target data rate	≥500 bps

**Table 2:** MCS table: SINR versus RR required to achieve R = 500 bps.

RR Per Node	SINR Range (dB)
1	[–20.6; +∞]
2	[–22.6; –20.6]
3	[–23.6; –22.6]
4	[–23.7; –23.6]
5	[–23.9; –23.7]
6	[–25.1; –23.9]

**Table 3:** The percentage change $\Delta m_{N}/m_{N,s}$ of the theoretical values $m_{N}$ and the empirical values $m_{N,s}$.

$\lambda_{b}$	SISO	1×8 SIMO	1×8 SIMO	1×8 MU-MIMO	1×8 MU-MIMO
		Selection	MRC	$n_{u}=2$	$n_{u}=4$
0.1	0.0012	0.0120	0.0417	0.0157	0.0392
0.3	0.0395	0.0435	0.0239	0.0144	0.0050
0.5	0.0201	0.0041	0.0398	0.0208	0.0916
0.7	0.0006	0.0080	0.0070	0.0206	0.2001
0.9	0.0206	0.0593	0.0292	0.0757	0.2811

**Table 4:** Statistical individual OFF probability with regard to antenna configuration, transmission mode and collector intensity $\lambda_{b}$.

$\lambda_{b}$	SISO	1×8 SIMO	1×8 SIMO	1×8 MU-MIMO	1×8 MU-MIMO
		Selection	MRC	$n_{u}=2$	$n_{u}=4$
0.1	23.40%	5.62%	**1.01%**	1.40%	2.90%
0.3	7.13%	0.31%	**0.03%**	0.07%	0.27%
0.5	3.97%	0.09%	**0.02%**	0.05%	0.20%
0.7	2.84%	0.07%	**0.01%**	0.05%	0.20%
0.9	2.30%	0.06%	**0.01%**	0.04%	0.21%

**Table 5:** The power distribution with the different transmission modes in the typical cell, $P_{RR}=25/6$ mW, $\lambda_{a}=5.5$ nodes/${\mathrm{km}}^{2}$, $\lambda_{b}=0.5$ nodes/${\mathrm{km}}^{2}$.

Power	SISO	1×8 SIMO	1×8 SIMO	1×8 MU-MIMO	1×8 MU-MIMO
(mW)		Selection	MRC	$n_{u}=2$	$n_{u}=4$
Off	3.97%	0.09%	**0.02%**	0.05%	0.20%
$P_{\mathrm{RR}}$	90.76%	99.39%	**99.92%**	99.80%	99.17%
$2P_{\mathrm{RR}}$	2.81%	0.35%	0.04%	0.10%	0.40%
$3P_{\mathrm{RR}}$	1.11%	0.09%	0.01%	0.03%	0.12%
$4P_{\mathrm{RR}}$	0.10%	0.01%	0.00%	0.00%	0.01%
$5P_{\mathrm{RR}}$	0.20%	0.01%	0.00%	0.00%	0.02%
$6P_{\mathrm{RR}}$	1.06%	0.06%	0.01%	0.02%	0.09%

## Abbreviations

The following abbreviations are used in this manuscript:

OFDMA	Orthogonal Frequency Division Multiple Access
IoT	Internet of Things
PPP	Poisson Point Process
RR	Radio Resource
SISO	Single Input Single Output
SIMO	Single Input Multiple Output
MRC	Maximum Ratio Combiner
MU-MIMO	Multiuser Multiple Input Multiple Output
ZF	Zero-Forcing
LPWA	Low Power Wide Area
LTE	Long Term Evolution
5G	Fifth Generation
3GPP	3rd Generation Partnership Project
4G	Fourth Generation
TTI	Time Transmission Interval
CSI	Channel State Information
SINR	Signal to Interference plus Noise Ratio
NPUSCH	Narrowband Physical Uplink Shared Channel
RB	Resource Block
RRB	Radio Resource Block
MCS	Modulation and Coding
PMF	Probability Mass Function
PGFL	Probability Generating Functional
LLS	Link Layer Simulation
PUSCH	Physical Uplink Shared Channel
CDF	Cumulative Distribution Function

## Appendix A. Proof of Proposition 1

Consider the set of all active nodes $x\in \Phi_{a}$ generated by the homogeneous PPP, the node *x* is in the typical cluster $\Phi_{c}\left(0\right)$ if the origin is o is the closest point to *x* among all the other collectors $y\in \Phi_{b}$, i.e., $|x|<|y-x|,\;\forall y\in \Phi_{b}$. We can note that the following indicator function

(A1) $$\prod_{y\in \Phi_{b}}{𝟙}_{\left\{|x|<|y-x|\right)}=\left\{\begin{array}{cc} 1 & x\in \Phi_{c}\left(0\right),\\ 0 & \mathrm{otherwise}. \end{array}\right.$$

The average number of users in this typical cell is,

(A2) $$E\left[{𝟙}_{\left\{x\in \Phi_{c}\left(0\right)\right\}}\right]=\sum_{x\in \Phi_{a}}\left[\prod_{y\in \Phi_{b}}{𝟙}_{\left\{|x|<|y-x|\right)}\right]$$

Using Campbell Theorem,

(A3) $$\sum_{x\in \Phi_{a}}\left[\prod_{y\in \Phi_{b}}{𝟙}_{\left\{|x|<|y-x|\right)}\right]=\int \left[\prod_{y\in \Phi_{b}}{𝟙}_{\left\{|x|<|y-x|\right)}\right]\lambda_{a}dx.$$

For a given $x\in \Phi_{a}$, we use the PGFL property on the PPP of $\Phi_{b}$,

(A4) $$\begin{array}{ccc} E\left[\prod_{y\in \Phi_{b}}{𝟙}_{\left\{r<|y-x|\right\}}\right]\;\;\; & = & \;\;\;\exp\left(-\lambda_{b}\int_{R^{2}}\left(1-{𝟙}_{\left\{r<|y-x|\right\}}\right)dy\right)\\ \;\;\; & = & \;\;\;\exp\left(-\lambda_{b}\int_{{\left|y-x\right|}^{2}<r^{2}}dy\right). \end{array}$$

Note that ${\left|y-x\right|}^{2}={\left|y\right|}^{2}+{\left|x\right|}^{2}-2|y||x|\cos\left(\theta_{z}-\theta_{y}\right)$ where |y| and $\theta_{y}$ (resp. r=|y| and $\theta_{x}$) are the polar coordinates of *z* (resp. *y*). The condition ${\left|y-x\right|}^{2}<r^{2}$ in (A4) is then equivalent to $\left|y|<2|x|\cos(\theta_{y}-\theta_{x}\right)$ with $0\leq (\theta_{y}-\theta_{x})\leq \pi$ to guarantee a positive range of $r_{y}$. The corresponding integral is then,

(A5) $$\int_{{\left|y-x\right|}^{2}<r^{2}}dy=\int_{0}^{\pi -\theta_{x}}\int_{0}^{2r\cos(\theta_{y}-\theta_{x})}r_{y}dr_{y}d\theta_{y}=\int_{0}^{\pi -\theta_{x}}r^{2}\cos^{2}\left(\theta_{y}-\theta_{x}\right)d\theta_{y}=\pi r^{2}$$

independently of *x*. Replacing in (A3), Equations (A4) and (A5), we have,

(A6) $$E\left[{𝟙}_{\left\{x\in \Phi_{c}\left(0\right)\right\}}\right]=\int_{0}^{\infty }\int_{0}^{2\pi }\exp\left(-\lambda_{b}\pi r^{2}\right)\lambda_{a}rdrd\theta =\frac{\lambda_{a}}{\lambda_{b}}.$$

Finally, the pdf of *r* can be deduced using the CDF of *r*,

(A7) $$\mathrm{Prob}\left\{x\in \Phi_{c}\left(0\right)\right\}=\mathrm{Prob}\left\{\prod_{y\in \Phi_{b}}{𝟙}_{\left\{r<|y-x|\right\}}\right\}=\exp\left(-\lambda_{b}\pi r^{2}\right)$$

## Appendix B. Proof of Proposition 2

The average number of radio-resources in the typical cluster is,

$$m_{N}=E\left[\sum_{x\in \Phi_{c}\left(o\right)}N_{RR}\left(x,A_{f},I,P_{n}\right)\right]=E_{\Phi ,\Phi_{b},A_{f},I,P_{n}}\left[\sum_{x\in \Phi_{a}}\underset{f(x)}{\underset{︸}{\prod_{y\in \Phi_{b}}N_{RR}\left(x,A_{f},I,P_{n}\right)\;{𝟙}_{\left\{|x|<|y-x|)\right\}}}}\right]$$

Using Campbell Theorem,

(A8) $$m_{N}=E_{A_{f},I,P_{n}}\left[\lambda_{a}\int E_{\Phi_{b}}\left[f(x)\right]dx\right].$$

with dx=rdrdθ and

(A9) $$E_{\Phi_{b}}\left[f(x)\right]=N_{RR}\left(x,A_{f},I,P_{n}\right)\;E_{\Phi_{b}}\left[\prod_{y\in \Phi_{b}}{𝟙}_{\left\{|x|<|y-x|)\right\}}\right].$$

Using the relationships (A4) and (A5),

(A10) $$m_{N}=\frac{\lambda_{a}}{\lambda_{b}}\;E_{I,P_{n}}E_{A_{f}}\left[\int N_{RR}\left(r,A_{f},I,P_{n}\right)\;2\pi \lambda_{b}r\exp\left(-\lambda_{b}\pi r^{2}\right)dr\right].$$

Notice that $2\pi \lambda_{b}r\exp\left(-\lambda_{b}\pi r^{2}\right)$ is nothing but the pdf of the smallest distance between the sensor and its nearest collector. This implies that,

(A11) $$m_{N}=\frac{\lambda_{a}}{\lambda_{b}}\;E_{r}\;E_{I,P_{n}}\;E_{A_{f}}\left[N_{RR}\left(r,A_{f},I,P_{n}\right)\right].$$

## Appendix C. Proof of Proposition 3

Considering the distance based scheduling algorithm, we adjust the dimensioning of each group with respect to the $i\times n_{u}^{\mathrm{th}}$ nearest neighbor to the typical collector ($i\in N^{*}$). We let $r_{i}$ denote the distance between this $i\times n_{u}^{\mathrm{th}}$ nearest neighbor $x_{i}=r_{i}e^{j\theta_{i}}$ and the typical collector. The average number of required RR in the typical cluster is the total number of RR required by this $i\times n_{u}^{\mathrm{th}}$ nearest neighbors, such that,

(A12) $$m_{N}=E_{r_{i},\theta_{i},A_{f},I,P_{n}}\left[\sum_{r_{i}:i\in N^{*}}N_{RR}\left(r_{i},A_{f},I,P_{n}\right)\prod_{y\in \Phi_{b}}{𝟙}_{\left\{r_{i}<|y-x_{i}|\right\}},\right]$$

By average on the collectors distribution as in Proof in Appendix B,

(A13) $$E_{\Phi_{b}}\left[\prod_{y\in \Phi_{b}}{𝟙}_{\left\{r<|y-x_{i}|\right\}}\right]=\exp\left(-\lambda_{b}\pi r_{i}^{2}\right).$$

The average number of required RR is then,

(A14) $$m_{N}=E_{A_{f},I,P_{n}}\left[\sum_{i\in N^{*}}\int N_{RR}\left(r_{i}\right)\exp\left(-\lambda_{b}\pi r_{i}^{2}\right)f_{R_{i}}\left(r_{i}\right)dr_{i}\right]$$

with

(A15) $$f_{R_{i}}\left(r_{i}\right)=\frac{2{\left(\pi \lambda_{a}\right)}^{in_{u}}}{(in_{u}-1)!}r_{i}^{2in_{u}-1}\exp\left(-\lambda_{a}\pi r_{i}^{2}\right)$$

being the distribution of $i\times n_{u}^{\mathrm{th}}$ nearest neighbor of the typical collector.

## Appendix D. Proof of Proposition 5

Using a single antenna receiver, the SISO fading coefficients are Rayleigh distributed and $A_{f}$ has an exponential distribution, and,

(A16) $$E_{A_{f}}\left[{𝟙}_{\left\{A_{f,k}\leq A_{f}<A_{f,k-1}\right\}}\right]=e^{-\left(P_{n}+I\right)s_{k}}-e^{-\left(P_{n}+I\right)s_{k-1}}.$$

Proposition 5 is obtained considering the average behavior of the noise and interference.

## Appendix E. Proof of Proposition 7

### Appendix E.1. Laplace Transform of the Interference with MRC

To show that the Laplace transform remains the same, we let $h_{x}=\left[h_{x,1}\ldots h_{x,n_{r}}\right]$ denote the complex Gaussian vector containing the (signal) fading coefficients and $h_{y}=\left[h_{y,1}\ldots h_{y,n_{r}}\right]$ the fading vector between the interferer and the collector. When using a MRC decoder, the $n_{r}$ observations of *x* are projected into the orthonormal vector $u_{x}=h_{x}/∥h_{x}∥$. The equivalent interferer channel is then the scalar product between $u_{x}$ and $h_{y}$. Let $u_{y}=h_{y}/∥h_{y}∥$, the equivalent fading seen by each interferer $A_{f,i}={\left|\langle u_{x},h_{y}\rangle \right|}^{2}$ is exponentially distributed. This is a consequence that the cosinus square of an angle between a Gaussian vector and a given direction $\cos^{2}\left(u_{x},h_{y}\right)$ is beta distributed with parameters 1 and $\left(n_{r}-1\right)$ as shown in [27]. This leads to a distribution of $\beta (1,n_{r}-1)$ multiplied by a chi-squared with $2n_{r}$ degrees of freedom which is exponentially distributed. In other words,

(A17) $$A_{f,i}=|\langle u_{x},h_{y}\rangle {|}^{2}={∥h_{y}∥}^{2}\cos\left(u_{x},u_{y}\right)∼\chi_{2n_{r}}^{2}\times \beta \left(1,n_{r}-1\right)=\mathrm{exprnd}\left(1\right).$$

### Appendix E.2. Fading Distribution

Using the distribution of $A_{f}$ in (37),

(A18) $$E_{A_{f}}\left[{𝟙}_{\left\{A_{f,k}\leq A_{f}<A_{f,k-1}\right\}}\right]=\sum_{p=0}^{n_{r}-1}\frac{1}{p!}\left[{\left(P_{n}+I\right)}^{p}s_{k}^{p}e^{-\left(P_{n}+I\right)s_{k}}-{\left(P_{n}+I\right)}^{p}s_{k-1}^{p}e^{-\left(P_{n}+I\right)s_{k-1}}\right],$$

By averaging over the noise and the interference level, Proposition 7 is obtained by invoking the following lemma:

**Lemma** **A1.**
*Let $\Xi_{i}\left(s\right)=E\left[s^{i}{\left(P_{n}+I\right)}^{i}e^{-s(P_{n}+I)}\right]$ Then,*

(A19) Ξi(s)=(−1)i∑j=0isiijdjLPn(s)dsjdi−jLI(s)dsi−j.

**Proof.** The expansion of $\Xi_{i}\left(s\right)=E\left[s^{i}{\left(P_{n}+I\right)}^{i}e^{-s(P_{n}+I)}\right]$ gives

(A20) Ξi(s)=1i!∑j=0isiijEPnjIi−je−s(Pn+I).

Due to the independence of $P_{n}$ and *I*,

(A21) Ξi(s)=1i!∑j=0isiijEPnje−sNEIi−je−sI.

Note that, for a given random variable *X*, we have,

$$\begin{array}{ccc} \frac{d^{k}}{d^{k}s}\left(L_{X}\left(s\right)\right) & = & \frac{d^{k}}{d^{k}s}\left[\int_{R}e^{-sx}p_{X}\left(x\right)dx\right]\\ & = & {\left(-1\right)}^{k}\;E\left[X^{k}e^{-sX}\right] \end{array}$$

 □
